# Overcoming Old in Age-Friendliness

**DOI:** 10.1080/02650533.2014.993949

**Published:** 2014-12-24

**Authors:** J. Lindenberg, R.G.J. Westendorp

**Keywords:** ageing, qualitative methods, identity, image, age-friendliness, social space

## Abstract

In this article, we explore views on an age-friendly space in the Netherlands by analysing the responses of older individuals (*N* = 54) in focus groups and by examining the perspectives around an age-friendly zone in the Netherlands, *Parkstad Limburg*. We found that a central issue in the wishes for living at a later age are adjustments to envisioned physical limitations that come with the ageing process; this includes adjustments to ensure safety, accessibility and mobility, in order to facilitate older individuals' efforts to stay engaged with the world around them. In their wishes, the older participants constructed ideal dwelling places that closely resembled a senior home, but at the same time they rejected wishing to live in a place that was identified as a senior home. We explain this paradox by the representation of such a space as being for old people, i.e. needy older individuals, which was not how the older participants wished to be identified. We conclude that the conception of age-friendly environments will have to face the difficult challenge of overcoming the association with old age, while simultaneously taking into account adjustments that signify and relate to the ageing process and that seem inescapably tied to oldness.

## Overcoming old in age-friendliness

What we are concerned with, then, is the long history of space, even though space is neither a ‘subject’ nor an ‘object’ but rather a social reality – that is to say, a set of relations and forms. This history is to be distinguished from an inventory of things in space (or what has recently been called material culture or civilization), as also from ideas and discourse about space. It must account for both representational spaces and representations of space, but above all for their interrelationships and their links with social practice. (Lefebvre, 1991, p. 116)

## Introduction

Age-friendliness has received growing attention in the last two decades. Local, national and international governing bodies have put the development of age-friendliness at the top of the agenda. The UN already placed age-friendly spaces into focus with its international plan on action for ageing (2002), and the WHO has followed suit in 2005 with the global age-friendly environments programme (WHO, 2007). The field of research on age-friendliness has expanded with analyses of age-friendly initiatives and conceptualizations of age-friendliness, and age-friendliness has received more substantial attention in environmental gerontology (Kendig, 2003).

As Lui et al. (2009) have noted, age-friendliness can be approached in various ways: by means of a bottom-up approach which focuses on measures enhancing the empowerment of older persons, or more top-down via approaches in which governance mechanisms are more central. The present article is closer to the latter approach. We explored older people's views on what aspects are essential for creating an age-friendly environment. First, we delve into a regional initiative of age-friendliness in the Netherlands, *Parkstad Limburg*, and we then secondly compare this initiative to the perspectives of older persons on what constitutes age-friendly living, based on focus groups with 54 participants. We analyse why age-friendly zones or living environments, such as *Parkstad Limburg* or a senior home, do fulfil many of the living and environmental wishes that older persons formulate, while at the same time they are not considered attractive places to live in. We argue that age-friendliness faces the difficult task of, on the one hand, providing a space that takes into account the consequences of the ageing process – which in that respect is in fact quite similar to *Parkstad Limburg* or a senior home – while, on the other hand, avoiding that this very space produces associations with an image of oldness and a social categorization of a needy older group, which is not considered attractive by older persons.

## A friendly ageing place in the Netherlands

In the Netherlands, a region in the south of the country called Parkstad Limburg (transl.: park city Limburg) is considered the prototype of a ‘grey’ (age-friendly) zone (Klinkers, 2004, p. 158). Seven municipalities brought this zone into being as part of a regional redevelopment initiative to stimulate the economic development of the region and to deal with population shrinkage in combination with an ageing population. The initiative was mainly a response to the regional population achieving higher ages while at the same time experiencing shrinkage in the number of youth. In Dutch, these two developments have been called ‘*vergrijzing*’ (lit.: greying) and ‘*ontgroening*’ (lit.: de-greening) (Gerrichhauzen & Dogterom, 2007, p. 32). At present, Parkstad Limburg comprises eight municipalities that are experiencing population shrinkage due to out-migration and an ageing population. In 1999, Parkstad was created as part of a collaborative effort to deal with public services, transport and housing. Currently, about 20% of the inhabitants of this region are above the age of 65, a percentage that lies far above the average of 12% in the Netherlands (Statistics Netherlands, 2013). The number of inhabitants of the region is projected to decline by about 17% by 2025 (van Dam *et al.*, 2006; Dreijerink et al. 2012).

The name Park city was deliberately chosen to advertise the green environment surrounding the cities as a major attraction of the area. The decrease in population size was mainly framed as an opportunity for change and for inspiring another form of growth [e.g. in titles of documents and names of projects, which presented ‘shrinkage as opportunity’ (Gerrichhauzen & Dogterom, 2007), or were entitled ‘the new growth is shrinkage’ (Latten & Musterd, 2009)].

The region has introduced several initiatives to enhance the cities' suitability for their older inhabitants. It is marketing itself as a kind of laboratory for innovative elderly care (Bontje, 2013). It has, for instance, adjusted sidewalks to make walking with walker easier, and the number of older care facilities is increasing to ensure that all of the inhabitants can find a suitable place to live. Other efforts that have been made to accommodate the older population in these municipalities include, for instance, the provision of care appropriate living, the appointment of elderly advisors and the formation of social neighbourhood teams. The latter teams try to engage older persons as volunteers to achieve ‘a coproduction’ (Dekker *et al.*, 2012, p. 13) of initiatives in which older persons and professionals work together and therewith increase the participation of older persons in the development and realization of activities (Engel, 2012). The region widely markets itself as anticipating the imminent changes that need to be made to deal with an ageing population.

The side effects of such an ageing region are also noticed; schools and shops are slowly closing down, as are other facilities, such as hospitals and local clinics. Gyms and sports organizations are disappearing or merging to ensure a sufficient number of members (Elzerman & Bontje, 2013). This seems to make the region less attractive for younger and middle-aged persons, but as a side effect it does seem to attract older persons to the region. As Latten and Musterd (2009) analysed from migration movements:

Clearly it is the care-needy older persons that come back to Parkstad Limburg, but the middle- and higher income classes just so leave the region and often even migrate across country borders. Evidently, the living environments within Parkstad do not adequately satisfy the demands of these population categories. (p. 12)

In 2012, around 100 inhabitants of one of the cities decided to demonstrate against the closing of yet another school. Demonstrators said that it ‘reduced their quality of life’ and led to a ‘ghost village’.^1^ In a similar way, a television programme on Dutch public broadcast reported on ‘the shrinkage that gnaws’ and shot images of empty streets.^2^ On the one hand, we thus see an older generation returning to the region and a region that takes the initiative to meet this older generation's needs, while on the other hand the relative and absolute decline of the younger generations in the region results in a less varied supply of facilities and leaves younger and older inhabitants dissatisfied with the consequences of an ageing population.

The tension that is evident in making a place attractive over the life course is not exceptional. The imagined wishes of different age groups result in demarcated spaces through which places become marked as for one particular generation. These markings coincide with age-identities that signify a certain social categorization. The development of an age-friendly space is therefore as much related to the physical construction and organization of that space as to the social signification of these kinds of places. This has important implications for the age-friendliness of these places. The question logically is: are these imagined age-friendly spaces as age-friendly as assumed? In this article, we explore the embedded meaning of age-friendly spaces and the consequences of these meanings.

## Methods and data collection

We explored the ideas about age-friendly spaces based on data collected in eight focus groups in four cities in the Randstad region of the Netherlands. The focus groups were held in a preliminary phase of a combined qualitative and quantitative research on the ambitions and wishes of older persons. This research was conducted as part of the VITALITY! Programme of the Medical Delta (Medical Delta, 2012), a research-driven cluster of life sciences and technology in the West of the Netherlands.

In total, 54 older persons aged 55–85 participated in the focus groups that were divided into age groups 55–70 and 70+. Altogether, 26 females and 28 males participated. The average age was 70. The groups convened in the period January–February 2013 and were organized with the help of Trendbox, a strategic research agency that assisted in the recruitment of participants and the guidance of focus groups. The focus groups met for about 2.5 hours each. The topics discussed included ageing, social relations, daily activities, living, work or any form of volunteer work, and self-management. Responses were elicited by means of open questions, hypothetical scenarios and associative assignments.

All participants were informed about the purpose of the focus groups and signed an informed consent form. The participants received a fee of 50 euro each and were reimbursed for travel costs.

The meetings of the focus groups were recorded on DVDs. All responses were transcribed verbatim and then coded according to the grounded theory approach (as developed by Glaser and Straus (1999) [1967]) in the qualitative software programme NVivo.

## An age-friendly living

On the basis of the responses elicited in the focus groups, we analysed what the participants considered an age-friendly place. Our analysis started with wishes concerning their physical environment in terms of housing.

When asked what kind of space they would prefer and would find appropriate for living in as they became older, most participants stated that they would like to stay where they were currently living. Some conceded that in the future they would need to make adjustments. These adjustments were mainly based in an imagined future physical decline and bodily and mental conditions:

Male, 69: wider doors, and the thresholds should be removed, so that if people age, yes, and they experience difficulty with walking, that they can walk normally in their house and do not have to cross an enormous threshold.

Like numerous other participants, this man envisioned a future in which mobility problems would become the major determinant for what would make an appropriate space for aging. Some of the participants had already moved to some form of adjusted living. A female aged 66, for instance, had just moved to an assisted living facility, for which she had been on the waiting list for more than 10 years. The new place, most importantly, was close to shops and on the ground floor. She further motivated her move because her only son was living in the USA and she did not wish to ‘be dependent on him’.

In terms of living space, the wishes were quite unequivocal: most of the participants felt that with ageing, mobility problems would occur, and therefore living wishes followed this anticipation. Accessibility and mobility should be ensured, for instance, by means of a house without stairs, without thresholds, with automatically opening doors and with elevators.

The participants' wishes evoke an age-friendly space in terms of housing that closely resembles the kinds of places that Parkstad Limburg is trying to develop. The care-appropriate living arrangements and social neighbourhood teams of Parkstad Limburg would fit exactly with the wishes of the participants in this research, and therefore, looking at the wishes thus formulated, it would be an ideal place to live.

## An age-friendly surrounding

The centrality of the anticipated immobility and physical decline was further emphasized in wishes about the surroundings of an age-friendly home. The environment should accommodate the decreased mobility in providing a safe and accessible surrounding. Very much in line with the strategy of the region Parkstad, many older participants said that they wished to live in a green environment. When asked in one of the hypothetical scenarios to develop a whole new planet, some participants imagined environmentally friendly places that were self-renovating. Houses would be covered with paint that would regenerate itself, and windows would be automatically cleaned by some self-managing mechanism.

Around the house, participants envisioned several ways to enhance mobility. They discussed ways to improve accessibility for persons with walker and in wheel chairs, which included adjusted sidewalks similar to those in Parkstad Limburg, or special tracks for walker. Some also imagined new forms of transportation, such as flying or self-driving cars, or new ways of using old forms of transportation, such as sharing segways or scooters.

Male, 69: then I would like to have an elevator, like such a small [chair] elevator. Flying cars for transport, lots of green around me and everything on the ground floor. A shopping mall and health care institute around the corner.

Important in this respect was that these were all solutions to ensure easy access. Closeness to facilities like a shopping mall, a supermarket or a hospital also was key in the ideas about what an age-friendly place should look like.

Male, 78: A living park, a community, where there aren't any cars, but where all facilities are close at hand. Pathways where you can drive with your scooter. All recreation and care all within one's reach and alternated with green parks.

Safety was also an essential aspect of an age-friendly environment, and some participants proposed a kind of community living with limited access for outsiders, preferably with a self-closing or automatically closing lock, where people pay attention to each other and keep an eye on one another. This was a wish for some participants, mainly because they felt that resilience and coping would decrease as a result of physical decline with advancing age, and consequently they expected their ability to deal with aggression or surprises to decline as well.

## Ageing and age-friendliness

These sketches of what would be an age-friendly space for living and age-friendly surroundings are very much related to the indeterminate and at times ambivalent ideas about ageing and what it means to be old, or rather what it means to be growing old. As is clear from the descriptions above, predominant aspects in the conception of age-friendly environments were ways to preserve mobility and to increase accessibility.

This focus on mobility and accessibility seems related to and determined by how participants viewed the ageing process and the social and societal implications of being old. When discussing ageing, many voiced that they did not feel old. One male participant aged 84 years said: ‘what is getting old? Old is how you feel. I do not think about the fact that I am 84 years old.’ A similar statement was made by a 70-year-old man: ‘I say I am old. But that is what others ascribe to me. It is not how I feel, nor what I feel.’

Thus, for many of the participants, being old seemed to be a distant future. What is more, the large majority felt that for themselves they would not count as being old. Even though some of them did have the chronological age to be socially seen as old, and they themselves felt that they were treated as being ‘old’, they distanced themselves from this age identity. A participant aged 84 who said he did not feel old later said:

That I can do less, I accept that, because that is in the end what getting older is. […] That I have to put on my glasses if I wish to read, that is not an issue for me, then I simply put the things on. You can take things difficult or easy. I find the only important thing in getting older is health. That is your capital.

The importance of health as a means to fulfil wishes and ambitions was crucial for many participants. Many did already experience health problems and declining mobility and felt that they were ageing: ‘well, sometimes I do feel old, but that's mainly because of the things I am no longer able to do’ (male, 72 years old). Similarly, another participant said: ‘you become physically weaker all the time, that's what bothers me’ (male, 70 years old). However, they did not see this as being old. Being old was something different, as one male participant aged 72 strikingly commented: ‘being old is being needy’. Although they realized that they were ageing, being old or perhaps *aged* was something different. Many participants therefore mainly saw being old as a distant future, irrespective of age and the experienced ageing process. It was less clear when and how one was to establish whether one was old, and the ambivalence and uncertainty in the timing seemed to influence decision-making on when a new place should be looked for. Sometimes, the imagined future was unpredictable. One man, aged 73, described how he and his wife had moved to a smaller, one-floor apartment in a large building with an elevator, as his wife had mobility problems after hip surgery. Now, about 1.5 years later, however, she was well able to walk again, and he felt that the move could have been postponed for some time at least.

The assumed future of being old rested mainly on a certain concept of what the ageing process entails. In this conceptualization, the most significant aspect was continuous physical decline, which mainly undermines both personal and societal perspectives on what it means to age well:

Female, 66 years: I mean, there is a gentleman who lives upstairs from me who is 91 years old and he has this kind of chair elevator, well and then you watch him go to the supermarket and going his way, I think that is absolutely great. That is what I have in mind. I don't know if I can grow old like that.

This female participant formulated an ideal in which the importance of being mobile and having access was mainly related to an ideal way of ageing, which would include remaining independent and having a degree of freedom to get around and make one's own decisions. The envisioned inherent physical dependence in ageing would then be compensated through an increase in accessibility and mobility aids. The striving to maintain the ability to get around was related to independence. This desire for independence was related to two aspects in ageing well, first of all an engagement with society, but, second, it also projected assumed social values according to which being dependent on others was supposedly unacceptable. In one focus group, this was illustratively discussed:

Interviewer: Can you describe positive sides about ageing?

Male, 72 years: Well, old, that is an individual question. It might be positive for yourself, your generation, so to say. But for humanity there is nothing positive in ageing.

Female, 71 years: No, they [society] all find it negative because they have to pay for it.

Male, 84 years: exactly.

Male, 70 years: a nice example of how old people are treated is uh when you [the interviewer] saw us, you open the elevator so the oldies can go up and then you triumphantly said ‘I'll take the stairs’.

Interviewer: I thought it was a bit full … […]

Male, 70 years: But that is an example of, of the way people look at older people.

Interviewer: and that is? How would you describe that?

Male, 70 years: having a bad leg and not capable of climbing the stairs.

Female, 74 years: I think that you have complainers; I mean people look upon them like ‘yuck’.

Evidently, some of the older participants did not see the interviewer's well-intended focus on the possible physical limitations of the participants as a sign of respect or courtesy, but rather as an implicit reference to their dependence and, by extension, to their being a burden to society. Interestingly, the cheerful tone the interviewer had used to indicate that taking the stairs was no burden at all had not mitigated the participants' negative interpretation, but seemed to confirm their assumption that younger people ‘triumph’ over the physical limitations of older people. This incident demonstrates the rather contradictory feelings that the ageing process can involve: a physical decline that leads to dependence and causes feelings of uncertainty and inferiority, and at the same time a powerful desire to place a strong emphasis on being capable and independent. Ageing, here seen as simply enjoying a long life, was seen as something desirable, but old was something that these participants did not feel they were or would become in the near future. This determined their wishes for an age-friendly environment, which on the one hand should accommodate their wishes for a safe and adjusted place, while at the same time facilitate independence without drawing attention to dependence.

The wish for independence was related to being capable and having freedom, but also to societal engagement. Being independent and mobile meant getting around and being able to play a role in society. A large majority of participants also discussed other forms of living that would ensure some ability to ‘stay close to the world’ (female, 73 years old). Many proposed a form of intergenerational living and wished for a neighbourhood in which several generations would live together. One participant aged 71 described how he had moved from a quiet rural village to a smaller, one floor, apartment in an apartment block in the centre of the city. He motivated his move saying that in the rural village, ‘everything disappeared, shops, facilities, and liveliness’. He had therefore chosen an apartment with a view to the street, to ensure that he would be able to enjoy the liveliness on the street.

Similar to this statement about ‘liveliness’, intergenerational living was seen as a way to remain engaged and stay part of society. As one male (74 years old) described:

The feeling, the feeling is, I guess, a question of living in mutuality. I believe that if, if … for people themselves it does not really matter if they are young or old. But if you no longer position yourself in society, I believe that you kind of become lonely, you implode, so to speak, you get pushed in, so to speak, and reach out less and less. So, I believe that the reciprocity between me and society, between me and what is going on around me, what is lively, that it should remain.

Independence and, similarly, living close to facilities and other generations were ways to remain engaged and were considered crucial in ageing well. Thus, many participants indicated that engaging with younger generations meant ‘being able to experience the future’ (male, 84 years old) and staying engaged in the present. While discussing the ideal place to live, participants formulated this as follows:

Male, 74 years: I believe that everyone should be able to live there [on this planet]. As it is an ideal environment. That [age] should not matter, if you isolate yourself as a group, I do not believe that is a good thing. You have to have input of all age groups. It must be a reflection of the normal world, because it is ideal, so we won't harm each other.

Male, 81 years: yes, such variety keeps you young, indeed.

Male, 74 years: otherwise, you get a society within a society.

This was also related to the expected physical decline, as physical limitations were seen as limiting the scope of engagement: ‘when you grow old, your perspective on the future becomes limited, your world becomes smaller’ (female, 71 years old). Ageing well is thus very strongly determined by the physical space, and particularly by what this space does or does not enable its older inhabitants to engage with.

## A dilemma for age-friendly spaces

Discussing an ideal, imagined age-friendly space, the ambivalence between, on the one hand, physical decline and wishes for accommodating the anticipated consequences of this decline and, on the other hand, wishes for continued societal engagement and for aging well without being old or being perceived as old. The dilemma this poses became especially clear in a discussion during one of the focus groups. When asked to imagine a planet on which the participants could design their own role for the older population, they were allowed, in a first phase, to simply brainstorm. In a second phase, some limitations were added, such as the absence of home care or the absence of a state pension. The following focus group participants were posed with the scenario of how they would organize themselves on a planet without state pension:

Male, 55 years: I can only think of one thing, I would still go with…, we go up there [the planet] with a whole group.

Interviewer: Yes?

Male, 55 years: I would after all, eh, go live together with a whole group to support each other.

Female, 69 years: yes.

Interviewer: yes, okay.

Male, 55 years: Yes, that's what I am thinking about anyway for the future. If you don't have any children, like me you know. So anyway in …, work together with a group, go live in a group and close to facilities.

Interviewer: and what would such a group look like?

Male, 55 years: young, old, everything all mixed up in the neighbourhood. Not too far apart from each other, but one should have one's own place on this planet, but well yes, helping each other a bit. In the neighbourhood, yes, yes. You should have a bit of freedom, also, also separate spaces in which you can come together again if you feel like it. Maybe there is also a doctor that uh …

Male, 56 years: Yes, we actually discussed such a thing for real, so definitely yes.

Male, 55 years: but in a group? And, that, that big?

Male, 56 years: yes, in a group. […] Well, so own spaces, you also have communal spaces. You have indeed, if necessary, your own care, eh within reach in the neighbourhood. […] Well yes, and then there should be an information committee, a group of people who feel like, well who wish to come and live in such a house.

Male, 55 years: kind of like a waiting list. A certain age, a waiting list.

Female, 56 years: in other words, it is a residential care facility.

Male, 55 years: no, that is not what one should call it, I also would, I would not want to live in a residential care facility when I am older.

Male, 67 years: well, who would want that, a residential care facility?

Female, 67 years: no one, right?

Male, 67 years: No.

In another focus group, one man aged 76 said something similarly contradictory in one and the same sentence when asked about the ideal planet: ‘I do think about a big house where all [old] people and facilities are in one place. I would like to live in my own house for as long as possible. Senior homes, I find them very limiting.’

Thus, while the participants constructed an age-friendly planet, they quickly arrived at a form of living that took into account the wishes as formulated above: safety and accessibility in an intergenerational neighbourhood. The trouble was, as the female aged 56 involved in the aforementioned discussion soon enough discovered, that what they were describing was quite similar to a residential care facility or a senior home. Right away they agreed that this is something none of them would want. It was quite obvious why they would not want to live in such a place, although it would fulfil all the conditions outlined earlier: residential care facilities are for ‘old people’, as the participants would say:

Female, 66 years: Well, I always say that I never want to live in a senior home.

Male, 69 years: No, all those old people.

On the one hand, some form of a senior home or communal living in which facilities and care are close by is considered an ideal space of age-friendliness, but on the other hand, the meaning attached to this kind of space as being designed for old people who are dependent and burdensome and do not contribute to society is something which only a few of the participants found acceptable. The explanation can be found in the ideas about what ageing well means: remaining independent and contributing to society. This leaves the necessity of widening one's physical reach, almost in a way to experience the world at your doorstep, as one becomes physically less capable of going out into the world. The dilemma is then that the anticipated physical trajectory seems to be best dealt with in some kind of special, segregated, adjusted environment, but this is undesirable because it would further limit the older citizen's capability to engage with the world. The ageing process itself, which is characterized by an increase of physical limitations, already limits the ability to engage with the world. As such, a special space that could be identified with being old and separate from society was undesirable. These kinds of senior homes were considered incompatible with the older participants' desires, because their identification with being old also meant that other age groups would not want to live there.

## Old-age-friendliness undermines age-friendliness

The conundrum then lies in the paradox that creating an age-friendly space means making certain adjustments that result in the representation of this space as old-age-friendly and not simply age-friendly. As Lefebvre (1991 [1974]) already noted, space produces and exerts certain power relations and social relations, and it is ‘a social reality’ and ‘a set of relations and forms’ (p. 116). The way we construct age-friendly spaces, and space itself, reproduces but also produces societal forms and social relations. Therefore, producing space also means producing a certain existence, creating spatial practices that are at the same time representational of space (following Lefebvre, 1991 [1974]). This becomes especially clear from this example of age-friendliness, where the construction of an age-friendly space, even by older persons themselves, produces a space and starts from spatial practices that socially signify an old-age-friendly space, which is considered unattractive. The very notion of old is incompatible with a desirable existence or identity, given the representational codes at the present time, and therefore an age-friendly place becomes an age-unfriendly place.

If we compare the characteristics of an age-friendly place as formulated by our participants with the physical characteristics of places such as Parkstad Limburg, this would be a very attractive region, considering that the initiatives developed fit our participants' wishes for an adjusted living place very well. At the same time, the decrease in other facilities and the declining presence of other generations conflict with another important wish of our participants, namely intergenerational environments that enable engagement with the world and experiencing the future. Age-friendly spaces that actually service for the possible consequences of physical limitations would fit the living wishes of our older participants very well, but exactly because they do this, they become associated with care-needy older persons, with whom few ageing persons wish to be identified. Moreover, the side-effect of the closing of facilities such as in Parkstad Limburg result in a further decrease of the young, as the lack of diversity in facilities have led to a flight of other generations (such as enterprises, commercial venues and schools). Paradoxically, this kind of age-friendliness makes a space less attractive for the older individuals who are the primary target of these age-friendly regions. Consequently, constructing an age-friendly space is conditioned by the social identification of older persons and the social categorization of being old. In fact, for our older participants, age-friendliness was determined by the degree to which age-friendliness did not become old-age-friendly and remained socially inclusive. The image of being old and the identification with being a needy elderly as embedded in a certain space (as for instance in Parkstad Limburg) are something which our participants viewed for others, and by moving to such a region they would become this other whom they preferred not to identify with.

The paradoxes and contradictions identified in this article, especially the representation of a space as old and the wish for an age-friendly, but not an old-age friendly space, resonate in some ways with the findings in other studies on spaces that cater for an older population, while they divert from them in other ways. Studies focusing on retirement communities that cater exclusively for older individuals, especially in the USA, argue that these communities, such as Sun City, represent a ‘retiree identity’ (Laws, 1995) based on ‘images of active affluent seniors enjoying their “golden years”’ (McHugh, 2000, p. 109), who wish to separate themselves from other generations (McHugh, 2007). Kastenbaum (1993) argues that, similarly, these communities provide retirees with a safe place separate from imaginary and non-imaginary threats in the outside world, or, as Kastenbaum (1993, p. 179) states, ‘the evils that threaten to engulf them’. According to Kastenbaum, this is part of a ‘fortress mentality’ (p. 167), a wish for a safe place that enables elders to be ‘encrusted’ (1993). These findings seem to diverge from our findings for an intergenerational space that engages and involves older persons in society. However, the emphasis on a safe place as found especially by Kastenbaum (1993) does resonate with the ideas of our participants. Part of the explanation for the divergences might be that the US retirement communities are explicitly putting forward a positive, affluent image of older age. This image comes close to what in the Netherlands would be called the *Zwitserleven* (trans.: Swiss life) experience, named after a marketing campaign of an insurance company that advertises its personal pension plan with images of a secure, affluent retirement in a holiday-like atmosphere. This positive image was not what our participants had in mind when they described an ideal age-friendly space, instead the space they described was for older individuals who were in need of certain help and therefore were provided with a safe, closed environment that was close to facilities and care and preferably without obstacles such as stairs or thresholds.

The intergenerational separation as identified in the studies on Sun City is fraught with ambivalence, as research showed that inhabitants do miss their grandchildren and cherish intergenerational contacts (McHugh, 2007), but at the same time the separation does provide a form of exclusiveness and safety to escape ageism and change, and creates an environment where ‘everybody is old, so nobody is old’ (McHugh, 2007, p. 296). This latter rationale actually comes very close to why our older participants rejected an age-friendly space. It is as if these retirement communities offer different solutions for the same problem of escaping the social image and identification of oldness. Sun city provides an escape from an ageist world, in which older individuals cannot or do not want to engage because they feel that they do not have a position in this world and are treated as being needy and dependent, and as having an unworthy position in the society. Creating an attractive age-friendly space in this socio-cultural context is all about servicing for the physical limitations that come with ageing while rising to the challenge of ensuring that this space is not identified as an old space.

## Conclusion

We conclude that initiatives such as Parkstad Limburg focus on addressing expected physical limitations that come with the ageing process, and that these limitations are also central to our older participants' ideals about age-friendliness. Accessibility, safety and retaining mobility are important for older persons to ensure independence and societal engagement. This seems to fit the rationale that as the world becomes smaller – due to physical limitations and loss of social relations – the world should come closer to home.

On the basis of these wishes, older participants constructed an ideal which came very close to some form of adapted living, i.e. some form of senior homes, yet this is a form of living that most participants do not favour at all. In this article, we argued that although older persons themselves imagined some form of senior homes, this is not a desired form of living because it produces a social space that is demarcated as for people who are old. Being old is not a desired social identity and therefore these homes reproduce social relations and significations that are considered unattractive. As a result, in housing and environment, age-friendliness in the sense of accommodating for the consequences of the physical process of ageing is often old-age-friendliness and has the difficult task to overcome the implicit negative social production of the meaning of old.

## Footnotes

^1.^ See, for instance, http://www.hartvannederland.nl/nederland/limburg/2011/kerkrade-loopt-leeg/

^2.^ http://tegenlicht.vpro.nl/nieuws/nederland/krimpverhalen-limburgs-dagblad/de-krimp-die-knaagt.html
